# A New Rodent Model of Non-Alcoholic Steatohepatitis and Metabolic Syndrome

**DOI:** 10.5935/abc.20190219

**Published:** 2019-11

**Authors:** Fernando Gomes Romeiro, Lívia Alves Amaral Santos

**Affiliations:** Universidade Estadual Paulista (UNESP), Botucatu, SP - Brazil

**Keywords:** Obesity, Insulin Resistance, Metabolic Diseases, Liver Disease, Fatty Liver, Syndrome Metabolic, Inflammation, Stress Oxidative, Rats

Obesity is markedly linked to an increased risk of all-cause mortality and its prevalence has risen to unacceptable levels in the developed world.^1,2^ Obesity and insulin resistance compose the core of most cases of metabolic syndrome (MS), which is a group of conditions and traits associated with an increased risk of cardiovascular disease and diabetes (approximately 2-fold and 5-fold, respectively).^3^ Non-alcoholic fatty liver disease (NAFLD) is the hepatic manifestation of MS and has gained attention not only for obesity-related disorders but also for the progression to non-alcoholic steatohepatitis (NASH), liver cirrhosis, and hepatocellular carcinoma. Similar to the other metabolic components involved in MS, NAFLD treatment is based on lifestyle changes that are difficult to achieve in clinical trials, making preclinical studies an excellent option to increase the knowledge about the disease and to test the interventions proposed.

However, *in vitro* models have severe limitations to evaluate the hepatic and extrahepatic findings of human NASH due to its multifactorial etiology.^4^ As a result, data obtained from animals are largely assessed, with a growing interest in the development of mice models. Even if many of these models do not develop steatohepatitis as the strict definition applied to human liver tissue, they are still an outstanding source of knowledge about NAFLD and NASH.

Rodent models need to mimic NAFLD regarding their development by diet-induced obesity, which is deemed the most common risk factor for NAFLD in humans.^5^ Above all, the diet given to the mice should resemble human diets regarding macronutrient composition, not depending on toxins and leading to obesity, insulin resistance and systemic inflammation.^6^

Some of the most used mice models to simulate NAFLD are the methionine and choline-deficient diet and the choline-deficient L-amino-defined diet, but both have been criticized because the animals suffer weight loss and do not develop insulin resistance.^7^ In addition, the high-cholesterol diet promotes only slight increases in liver weight, triglyceride levels and serum liver enzymes, at the expense of an unusual amount of dietary cholesterol (1%) compared to human diets.^8^

The Cholesterol and Cholate (CC) diet also require a high amount of cholesterol (1.25%). It induces inflammation, hepatocellular ballooning, steatosis and fibrosis over 6-24 weeks.^4^ Furthermore, additions of 60% fat can shorten the development of NASH to 12 weeks.^9^ The model also causes lipid peroxidation, serum lipids increase and oxidative stress. However, mice submitted to this diet lose weight, have low plasma triglyceride levels and do not develop insulin resistance.^10^

The high-fat diet (71% fat, 18% proteins and 11% carbohydrates) is another interesting option to simulate NASH, developing insulin resistance and panlobular steatosis, but it is highly dependent on the animal strain and the diet composition.^4^

The high-fructose diet promotes hepatic inflammation and oxidative stress but does not induce the hepatic findings of NASH when administered *ad libitum*. Therefore, it would be expected that a high fat, fructose and cholesterol diet would be an excellent option to induce NASH by combining all the main components of each models mentioned above. Nevertheless, some mice models fed with these components do not develop advanced liver fibrosis.^4^ Other models bring some advantages, but they are more expensive and involve more technical issues.

An additional option to the rodent models of NAFLD is now presented by Muniz et al., in which mice were fed with a high-fat diet composed by lard (20%), cholesterol (1%) and cholate (0.1%). The regimen given to the mice is similar to the CC diet, leading to dyslipidemia and severe liver damage as seen in the human NASH.^11^ The animals not only gained weight but their liver weight was marked increased as well, reaching 5% of the total body weight. The authors postulated that the use of cholate could have accelerated the metabolic disturbances induced by the diet. Of note, the total fat composed only 44% of the dietary energy content, similar to the amount consumed by obese people (43-55%). Also, the authors obtained significant results after 6 weeks, whereas similar studies needed 9-12 weeks to achieve their aims.

The histological analysis presented in the article showed pronounced steatosis and marked inflammation. New studies should be developed in order to evaluate if these findings would lead to liver fibrosis and other consequences of human NASH. For the moment, the model presented by Muniz et al.,^11^ is a fast and low-cost option to induce NASH in mice by using a diet that resembles the ones consumed by obese people. Hopefully, this model can bring new information on NAFLD, NASH and MS, increasing the current knowledge about their pathophysiology and allowing evaluating new treatments against these metabolic disorders.
